# Supplementary material for: Evaluation of Label-Free Confocal Raman Microspectroscopy for Monitoring Oxidative Stress In Vitro in Live Human Cancer Cells
Source: Antioxidants (Basel). 2022 Mar 17;11(3):573. doi: 10.3390/antiox11030573 (PMC8945565; doi:10.3390/antiox11030573)
Supplement: Supplementary file 1 [file antioxidants-11-00573-s001.zip › antioxidants-1624086-supplementary.pdf]

Supplementary Data for:

Evaluation of label-free confocal Raman microspectroscopy for monitoring oxidative stress *in vitro* in live human cancer cells

Jakub Maciej Surmacki [1,2,3,*], Isabel Quiros-Gonzalez [1,2,4,5] and Sarah Elizabeth Bohndiek [1,2,*]

[1] Department of Physics, University of Cambridge, JJ Thomson Avenue, Cambridge, CB3 0HE, United Kingdom

[2] Cancer Research UK Cambridge Institute, University of Cambridge, Robinson Way, Cambridge, CB2 0RE, United Kingdom

[3] Institute of Applied Radiation Chemistry, Lodz University of Technology, Wroblewskiego 15, 93-590 Lodz, Poland

[4] Animal Histopathology Core at IUOPA, University of Oviedo, 33033 Oviedo, Spain

[5] Redox Biology and Metabolism in Cancer, Instituto de Investigación Biosanitaria ISPA, 33003 Oviedo, Spain

***** Correspondence: J.M.S. jakub.surmacki@p.lodz.pl, Tel.: +48426313188; S.E.B. seb53@cam.ac.uk, Tel.: +441223 337267

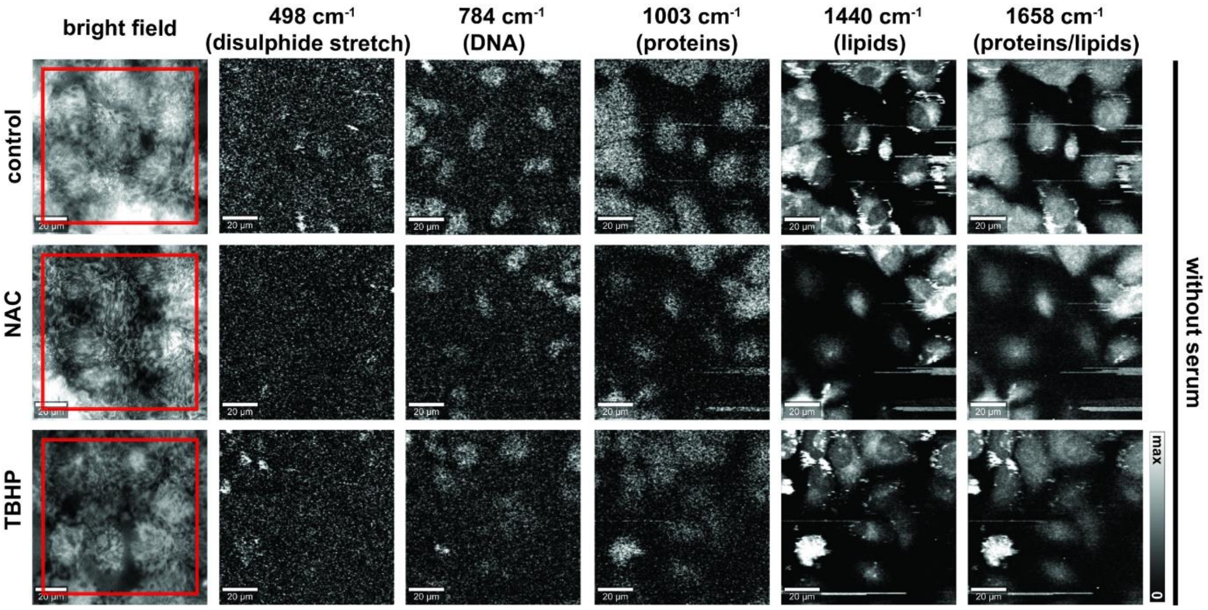

Supplementary Figure S1: Raman images of live A549 cells in media without serum prepared using different vibrational modes. Bright field, stitched bright field microscopy images. Raman images prepared to reflect nucleic acid content (784 cm^{-1}, sum filter: 772-796 cm^{-1}, scale: 0-150 cts), proteins (498 cm^{-1} (disulphide stretch), sum filter: 475-501 cm^{-1}, scale: 0-150 cts and 1003 cm^{-1}, sum filter: 991-1015 cm^{-1}, scale: 0-150 cts), lipids / proteins (1440 cm^{-1}, sum filter: 1425-1465 cm^{-1}, scale: 0-800 cts and 1658 cm^{-1}, sum filter: 1638-1678 cm^{-1}; scale: 0-800 cts). Final concentration of NAC was 1 mM and TBHP 200 μM. Images were acquired at 785 nm, with 0.5 s exposure at 120 mW. Spatial scale bar 20 microns. Small floating objects (e.g. cell debris or excretion) might generate streak artifacts (horizontal stripes) observed in some images due to the mechanical raster scanning of the Raman micro-spectroscopy stage.

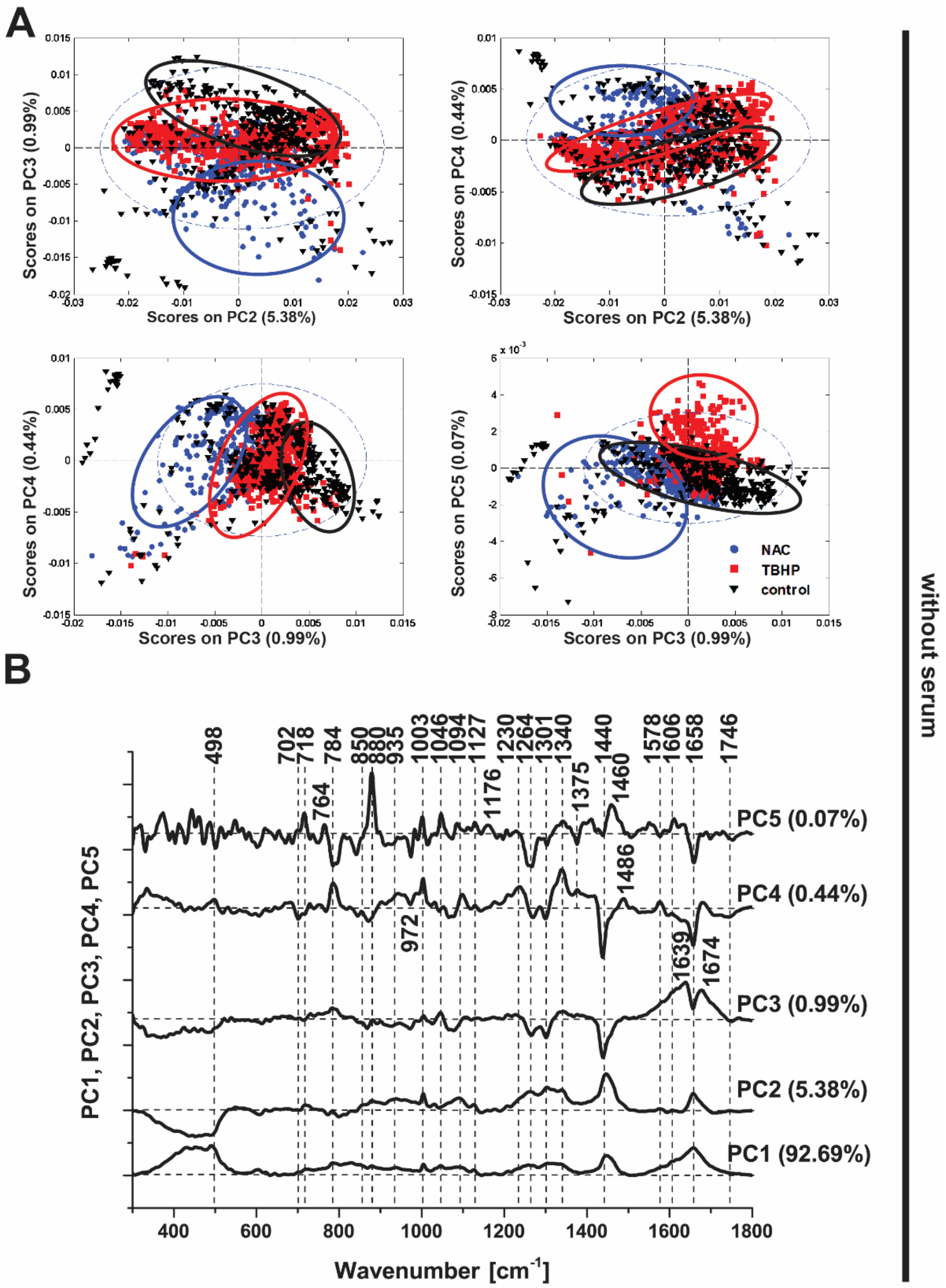

Supplementary Figure S2: Principal component analysis (PCA) of Raman spectra for cells in culture media without serum. (**A**) PCA scores plots (PC3 vs. PC2, PC4 vs. PC2, PC4 vs. PC3, PC5 vs. PC3). Scores plots show clustering of Raman spectra belonging to the treatment classes: NAC (light blue circle), TBHP (dark red square), and control (yellow triangle). (**B**) Loadings plot of PC1, PC2, PC3, PC4 and PC5 indicate the Raman bands that contribute to each principal component.

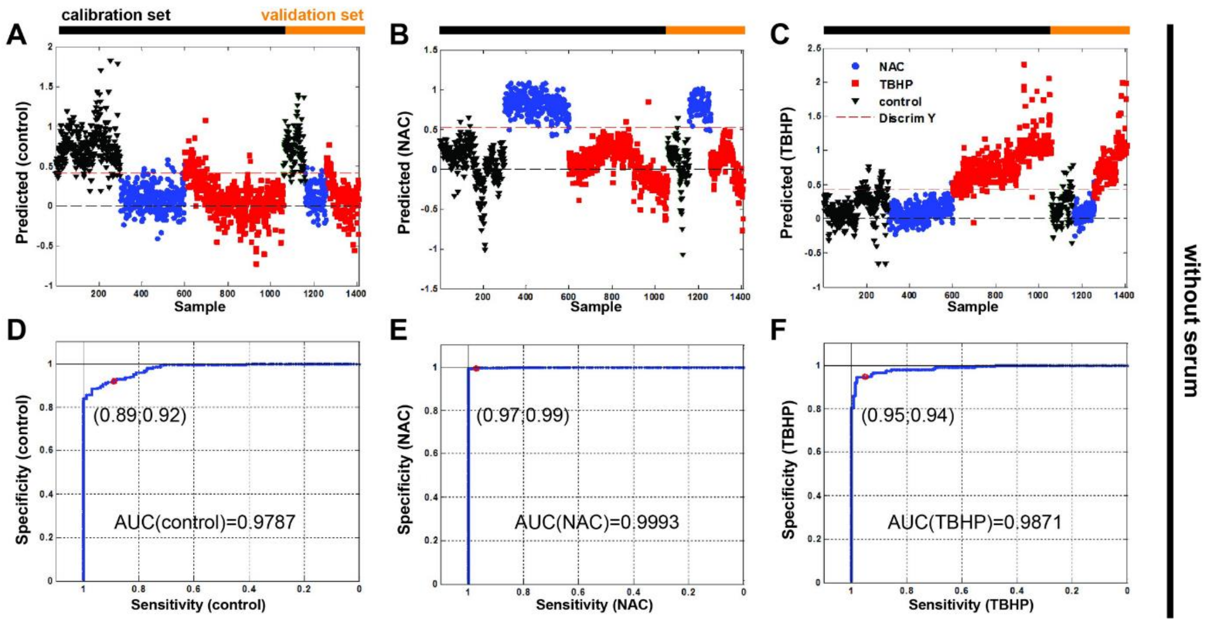

Supplementary Figure S3: Partial least squares discriminant analysis with receiver operating characteristic for cells in media without serum. The Raman spectra were split into sets for calibration [(**A**) n(control)=300, (**B**) n(NAC)=300, (**C**) n(TBHP)= 458] and validation [n(control)=100, n(NAC)=100, n(TBHP)=152]. Receiver operating characteristic (ROC) curves of all Raman data: (**D**) control, (**E**) NAC and (**F**) TBHP.

Supplementary Table S1. Kruskal-Wallis (K-W) ANOVA and Mann-Whitney (M-W) test of Raman band intensities between groups (at the $p<0.05$ level, the populations are significantly different for all Raman bands for data with serum by K-W ANOVA, whereas for medium without serum, bands at 1301, 1440cm^{-1} are not significantly different – marked as nsd).

Raman band position [cm^{-1}]	Raman band assignment	Sample	Condition with serum			Condition without serum			M-W
			median	Q1	Q3	median	Q1	Q3	p
498	S-S disulphide stretching	Control	**87.7**	70.1	124.2	**75.3**	59.6	97.3	3.08E-10
		NAC	**107.9**	84.8	128.5	**82.1**	71.5	103.3	2.00E-34
		TBHP	**75.2**	63.8	96.9	**73.6**	66.4	81.2	0.00167
		K-W ANOVA	p= 4.30E-72			p=3.00E-20			
718	CN^+-$(CH_3)_3$ symmetric stretching, phospholipids	Control	**25.6**	18.1	39.3	**21.3**	14.9	28.3	3.26E-11
		NAC	**23.9**	18.4	31.1	**18.9**	14.7	24.1	1.44E-19
		TBHP	**17.2**	13.7	22.5	**20.0**	13.1	27.6	1.10E-6
		K-W ANOVA	p=6.52E-64			p=0.01576			
784	Cytosine, uracil, thymine, pyrimidine bases, ring breathing modes in DNA bases	Control	**44.5**	31.7	62.0	**38.3**	29.4	51.8	4.70E-6
		NAC	**48.4**	39.3	59.5	**38.8**	30.2	47.0	1.67E-29
		TBHP	**36.6**	29.1	43.7	**33.7**	27.7	46.4	0.77484 (nds)
		K-W ANOVA	p=2.37E-60			p=4.83E-5			
880	Indole ring mode of tryptophan	Control	**31.0**	23.5	46.7	**27.9**	19.3	37.3	8.39E-7
		NAC	**28.9**	23.4	33.9	**23.9**	17.9	29.9	1.78E-15
		TBHP	**23.0**	18.4	28.6	**30.7**	21.8	59.0	7.34E-38
		K-W ANOVA	p=5.00E-56			p=1.42E-20			
1003	Phenylalanine, proline, symmetric stretching (ring breathing) mode of phenyl group	Control	**57.8**	35.7	78.3	**45.0**	22.9	60.8	3.39E-16
		NAC	**51.6**	36.9	61.0	**36.2**	23.4	46.2	6.45E-41
		TBHP	**37.0**	22.9	46.5	**39.0**	19.8	63.6	1.44E-6
		K-W ANOVA	p=7.04E-79			p=7.05E-7			
1094	Symmetric PO_2^- stretching mode of the DNA backbone	Control	**39.2**	23.9	54.2	**30.2**	15.8	43.1	1.72E-12
		NAC	**34.4**	25.1	41.5	**26.1**	16.0	32.6	1.57E-25
		TBHP	**24.3**	15.5	30.5	**27.4**	14.7	47.1	5.07E-11
		K-W ANOVA	p=8.06E-78			p=5.52E-5			
1264	=CH deformation,	Control	**50.6**	31.8	76.0	**35.8**	19.9	51.3	5.48E-18
		NAC	**41.5**	28.3	50.1	**31.8**	20.6	40.2	2.32E-17

	triglycerides (fatty acids), lipids	TBHP	**32.1**	20.2	40.8	**33.2**	15.3	54.2	0.01154
		K-W ANOVA	p=9.61E-59			p=0.01811			
1301	CH_2 twist, triglycerides (fatty acids), lipids	Control	**60.4**	40.1	85.8	**40.0**	22.5	57.7	2.70E-22
		NAC	**49.0**	32.9	61.0	**37.1**	25.4	49.2	8.17E-17
		TBHP	**38.6**	25.1	49.4	**38.8**	17.9	62.7	0.08513 (nds)
		K-W ANOVA	p=1.84E-60			p=0.210 (nds)			
1440	CH_2 and CH_3 deformations, lipids	Control	**81.9**	57.8	133.9	**56.8**	35.5	80.3	3.13E-23
		NAC	**62.5**	47.6	86.8	**48.8**	36.6	69.5	2.19E-14
		TBHP	**54.3**	38.3	73.4	**55.7**	28.0	86.4	0.32613 (nds)
		K-W ANOVA	p=1.06E-48			p=0.062 (nds)			
1606	Tyrosine, phenylalanine ring vibration C=C bending, cytosine NH_2, protein	Control	**53.7**	43.5	62.6	**44.9**	37.6	53.2	3.03E-27
		NAC	**47.6**	40.7	51.0	**37.7**	31.8	41.9	3.63E-81
		TBHP	**40.4**	35.1	44.6	**43.0**	35.6	56.0	1.24E-14
		K-W ANOVA	p=3.20E-125			p= 2.53E-39			
1658	Amide I, C=O stretching mode, peptide linkage; C=C stretching, lipids	Control	**115.8**	91.8	157.4	**90.1**	67.8	111.0	2.33E-26
		NAC	**99.2**	80.1	111.5	**78.5**	64.4	91.1	9.78E-36
		TBHP	**84.2**	68.1	97.6	**86.5**	62.3	117.6	0.00342
		K-W ANOVA	p=1.90E-83			p= 2.92E-10			
1746	C=O stretching, ester group of lipids and phospholipids	Control	**8.2**	6.2	10.7	**7.1**	5.8	8.9	8.13E-7
		NAC	**8.0**	6.5	9.9	**6.8**	5.6	8.4	1.16E-12
		TBHP	**7.6**	6.5	9.3	**6.8**	5.7	8.0	3.56E-20
		K-W ANOVA	p=0.0065			p= 0.0080			

Supplementary Table S2. Partial least squares discriminant analysis confusion table of all Raman spectroscopy data.

MODEL RESULTS						
	Actual Class					
	with serum			without serum		
	control	NAC	TBHP	control	NAC	TBHP
Predicted as control	424	8	24	286	5	31
Predicted as NAC	9	439	74	2	295	1
Predicted as TBHP	28	10	510	12	0	426
**CV RESULTS**						
	Actual Class					
	with serum			without serum		
	control	NAC	TBHP	control	NAC	TBHP
Predicted as control	418	12	23	285	5	31
Predicted as NAC	8	431	79	2	295	1
Predicted as TBHP	32	14	506	13	0	426
**PREDICTION RESULTS**						
	Actual Class					
	with serum			without serum		
	control	NAC	TBHP	control	NAC	TBHP
Predicted as control	141	1	6	93	2	11
Predicted as NAC	1	147	18	1	98	0
Predicted as TBHP	10	5	178	6	0	141
